# Supplementary material for: Choice of 16S Ribosomal RNA Primers Impacts Male Urinary Microbiota Profiling
Source: Front Cell Infect Microbiol. 2022 Apr 21;12:862338. doi: 10.3389/fcimb.2022.862338 (PMC9069555; doi:10.3389/fcimb.2022.862338)
Supplement: Supplementary file 1 [file DataSheet_1.pdf]

Supplementary Material

1. Supplementary Figures and Tables

1.1. Supplementary Figures

A high-quality copy of all supplementary figures are available as separate files.

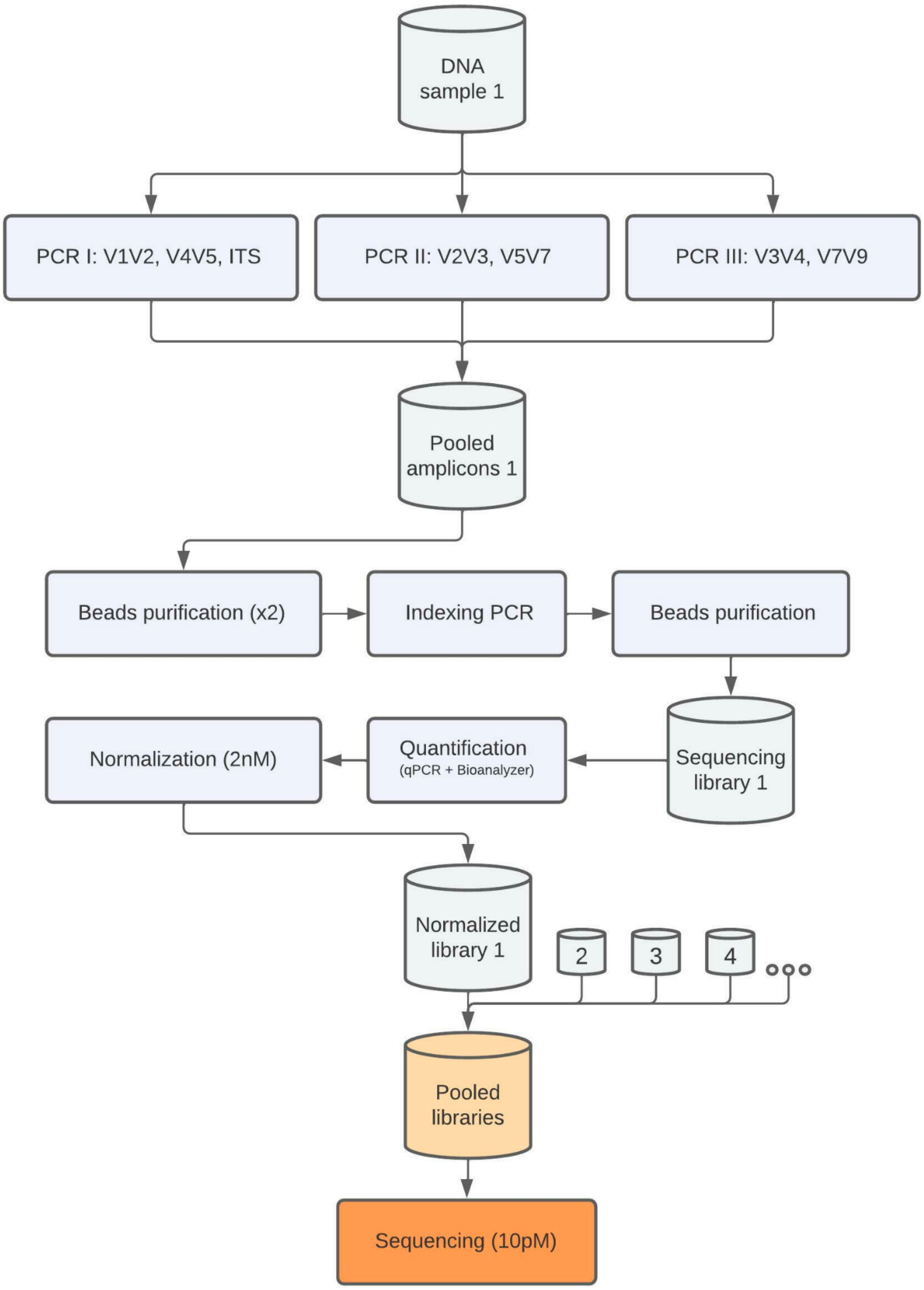

Supplementary Figure 1: Flowchart of key methods during sequencing libraries preparation.

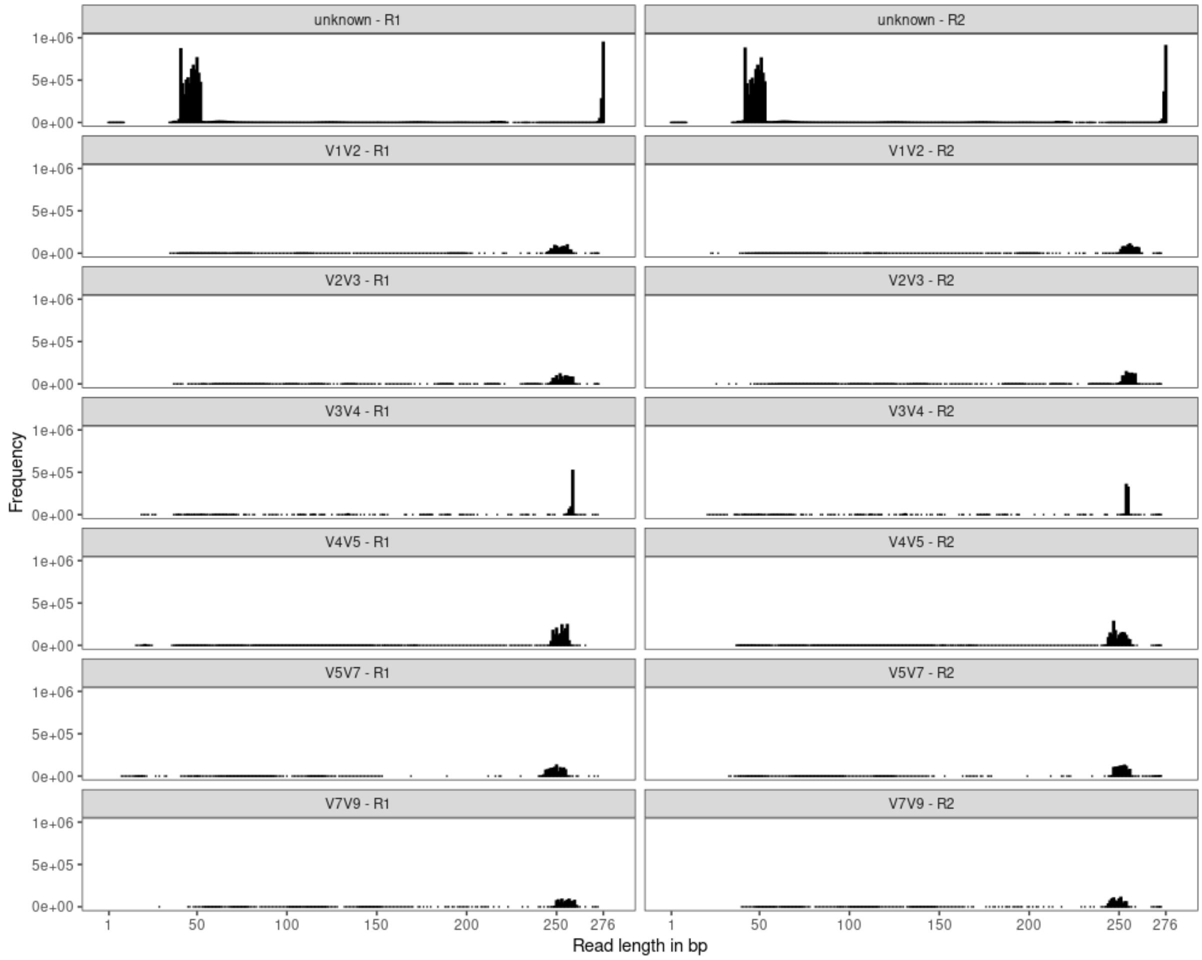

Supplementary Figure 2: Read lengths for each 16S rRNA amplicon-specific dataset. Histograms with distribution of read lengths in bp. Read lengths for sequences not associated with any of the amplicons of interest (unknown) are also shown. R1, forward reads; R2, reverse reads.

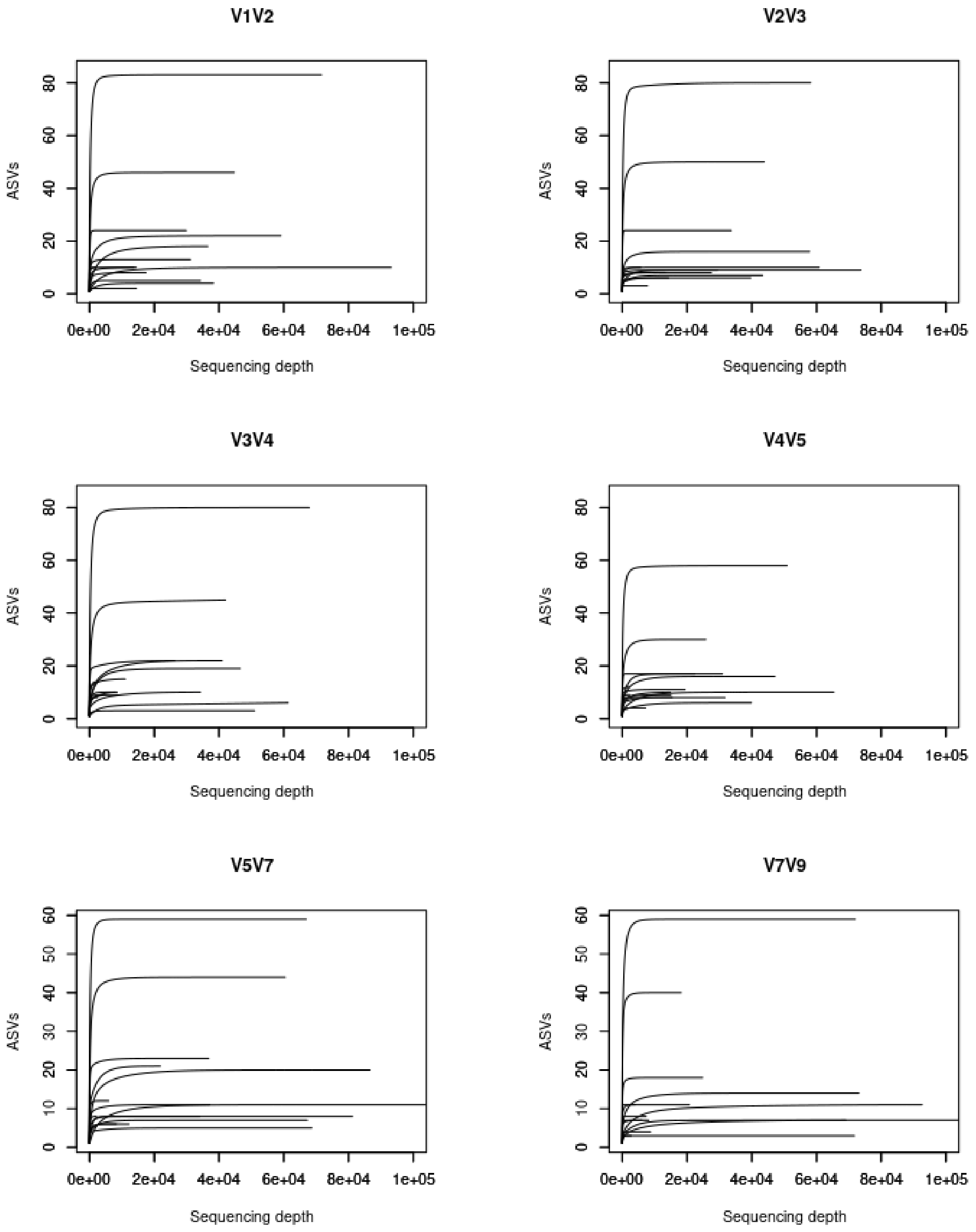

Supplementary Figure 3: Amplicon sequence variants (ASVs) rarefaction curves for each 16S rRNA amplicon-specific dataset. Each curve depicts a library. Reads were selected by random subsampling without replacement at incremental steps of 50 reads. Plots were truncated at sequencing depth equals 100,000 reads.

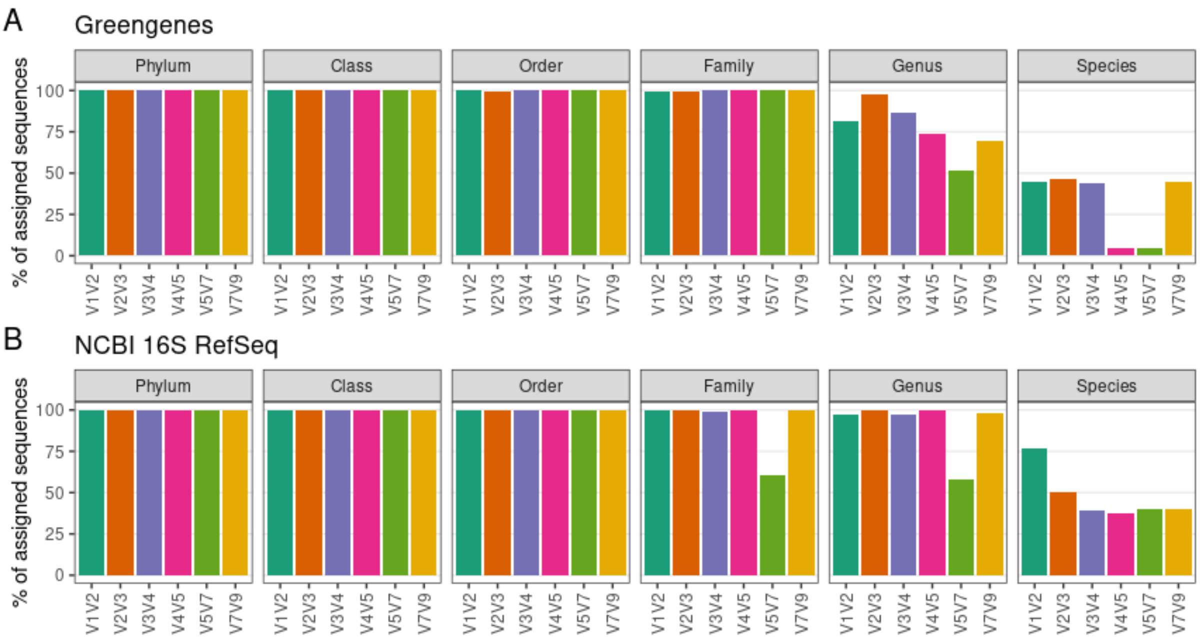

Supplementary Figure 4: Taxonomic resolution for each 16S rRNA amplicon-specific dataset. (A) Percentage of sequences with assigned taxonomy (per taxonomic level) for each amplicon-specific dataset when using Greengenes as reference database. (B) Percentage of sequences with assigned taxonomy (per taxonomic level) for each amplicon-specific dataset when using NCBI 16S RefSeq as reference database.

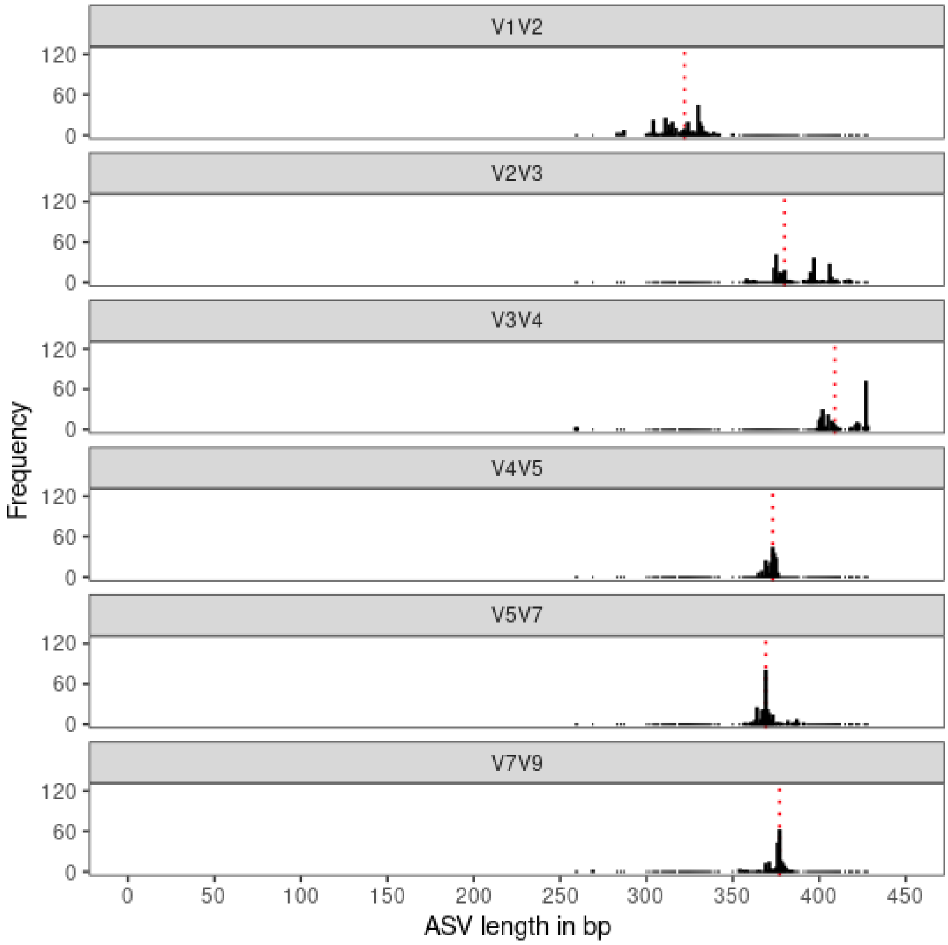

Supplementary Figure 5: Amplicon sequence variant (ASV) lengths for each 16S rRNA amplicon-specific dataset. Histograms with distribution of ASV lengths in bp. The median ASV size per amplicon-specific dataset is indicated by a red dotted line.

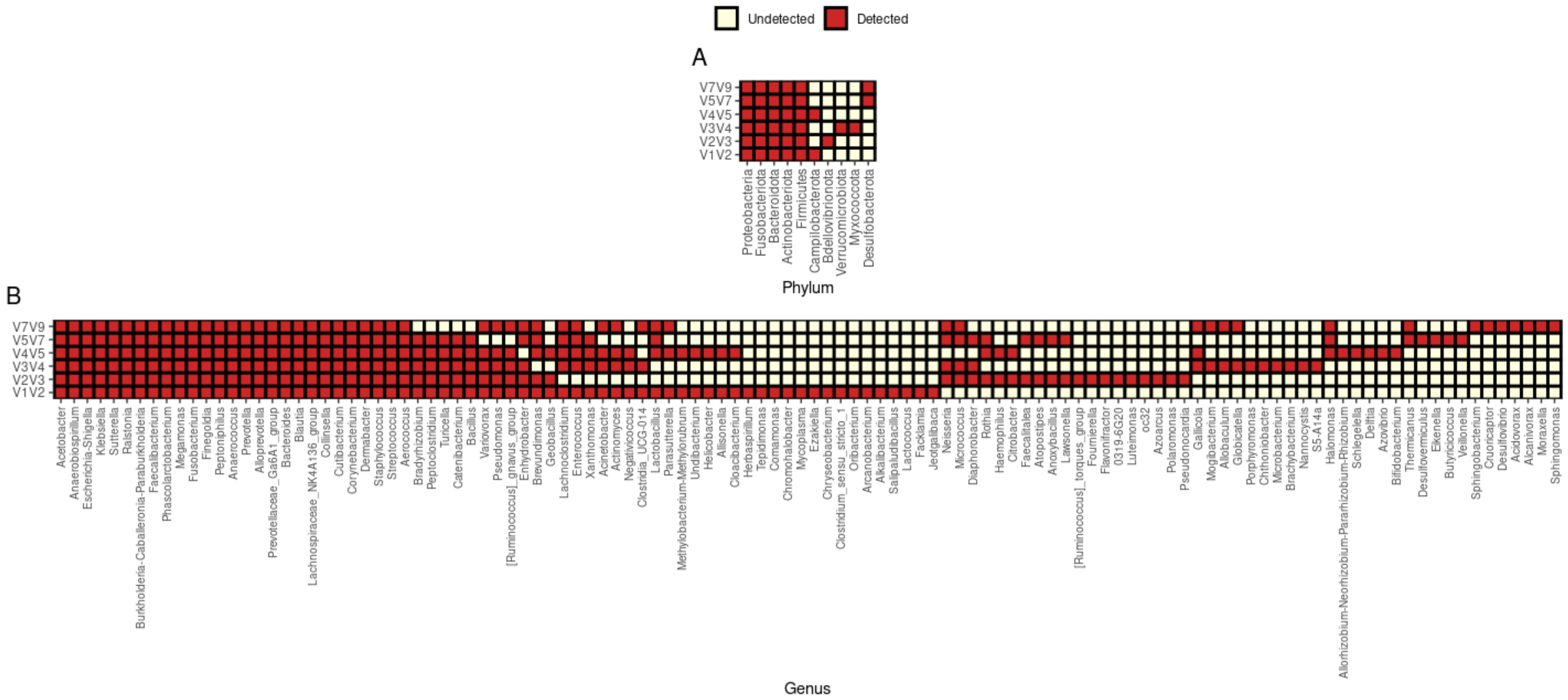

Supplementary Figure 6: Phyla and genera detected in each 16S rRNA amplicon-specific dataset. (A) Heatmap depicting the phyla detected in each amplicon-specific dataset. (B) Heatmap depicting the genera detected in each amplicon-specific dataset. Taxa are sorted based on the number of amplicon-specific datasets in which they were detected.

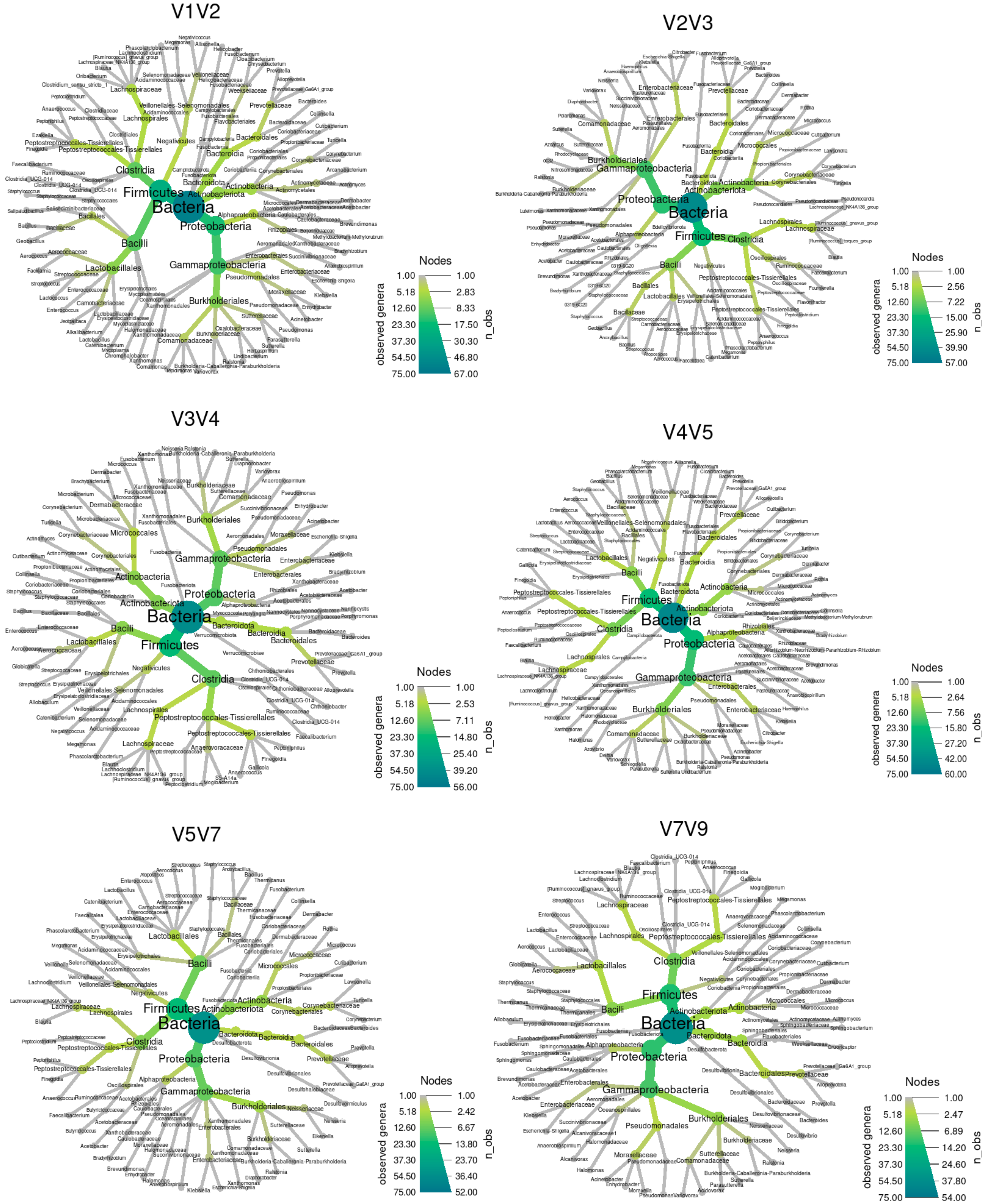

V1V2
Nodes
observed genera
1.00
5.18
12.60
23.30
37.30
54.50
75.00
n_obs
1.00
2.83
8.33
17.50
30.30
46.80
67.00
V2V3
Nodes
observed genera
1.00
5.18
12.60
23.30
37.30
54.50
75.00
n_obs
1.00
2.56
7.22
15.00
25.90
39.90
57.00
V3V4
Nodes
observed genera
1.00
5.18
12.60
23.30
37.30
54.50
75.00
n_obs
1.00
2.53
7.11
14.80
25.40
39.20
56.00
V4V5
Nodes
observed genera
1.00
5.18
12.60
23.30
37.30
54.50
75.00
n_obs
1.00
2.64
7.56
15.80
27.20
42.00
60.00
V5V7
Nodes
observed genera
1.00
5.18
12.60
23.30
37.30
54.50
75.00
n_obs
1.00
2.42
6.67
13.80
23.70
36.40
52.00
V7V9
Nodes
observed genera
1.00
5.18
12.60
23.30
37.30
54.50
75.00
n_obs
1.00
2.47
6.89
14.20
24.60
37.80
54.00

Supplementary Figure 7: Taxonomic tree of each 16S rRNA amplicon-specific dataset. Taxa detected in each amplicon-specific dataset are shown up to genus level (tree leaves). Node sizes and color scheme represent the number of leaves (number of genera) present in the subtree of the nodes. The Reingold-Tilford layout was used.

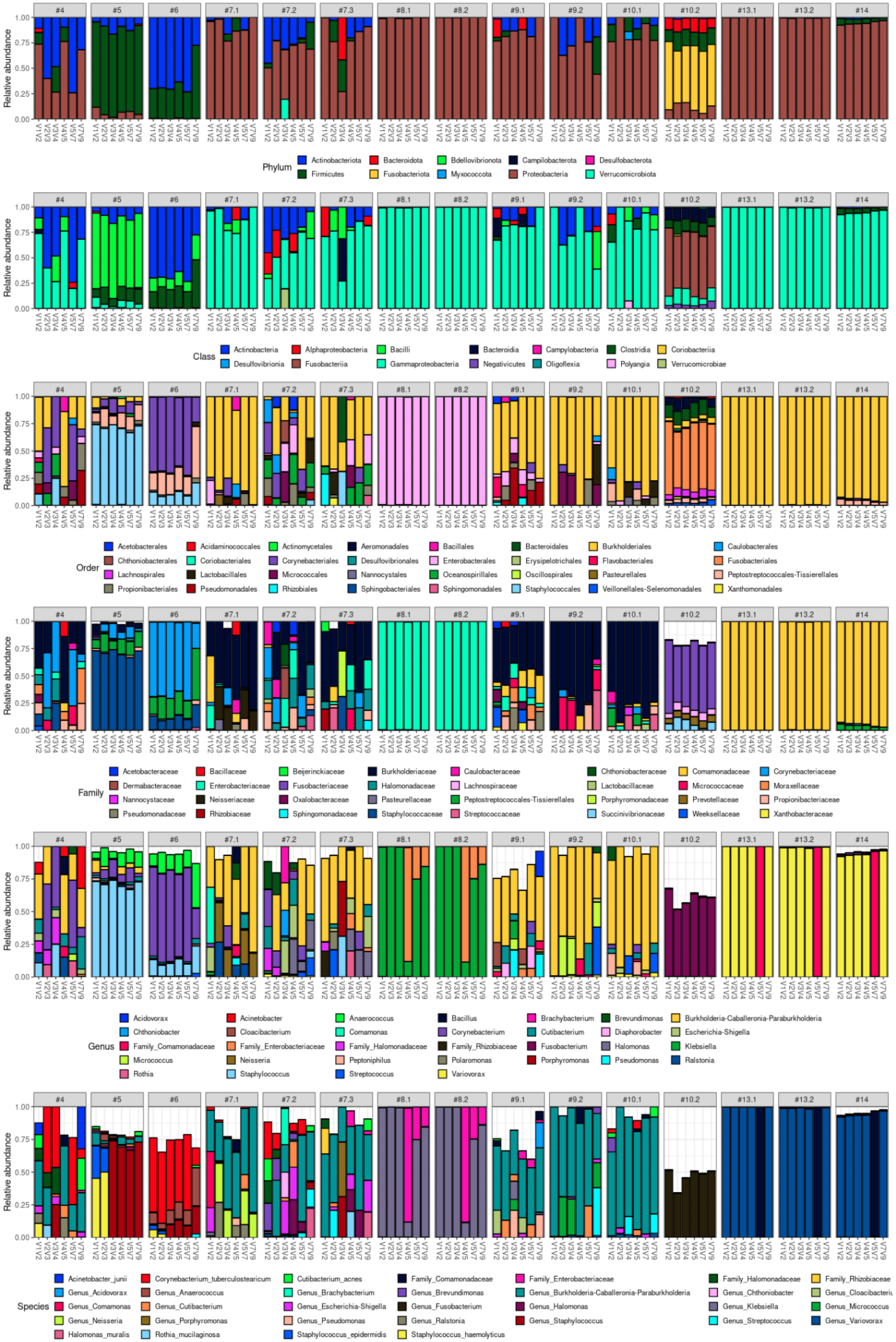

#4
#5
#6
#7.1
#7.2
#7.3
#8.1
#8.2
#9.1
#9.2
#10.1
#10.2
#13.1
#13.2
#14
Relative abundance
0.00
0.25
0.50
0.75
1.00
V1V2
V2V3
V3V4
V4V5
V5V7
V7V9
Phylum
Actinobacteriota
Bacteroidota
Bdellovibrionota
Campilobacterota
Desulfobacterota
Firmicutes
Fusobacteriota
Myxococcota
Proteobacteria
Verrucomicrobiota
Class
Actinobacteria
Alphaproteobacteria
Bacilli
Bacteroidia
Campylobacteria
Clostridia
Coriobacteriia
Desulfovibrionia
Fusobacteriia
Gammaproteobacteria
Negativicutes
Oligoflexia
Polyangia
Verrucomicrobiae
Order
Acetobacterales
Acidaminococcales
Actinomycetales
Aeromonadales
Bacillales
Bacteroidales
Burkholderiales
Caulobacterales
Chthoniobacterales
Coriobacteriales
Corynebacteriales
Desulfovibrionales
Enterobacterales
Erysipelotrichales
Flavobacteriales
Fusobacteriales
Lachnospirales
Lactobacillales
Micrococcales
Nannocystales
Oceanospirillales
Oscillospirales
Pasteurellales
Peptostreptococcales-Tissierellales
Propionibacteriales
Pseudomonadales
Rhizobiales
Sphingobacteriales
Sphingomonadales
Staphylococcales
Veillonellales-Selenomonadales
Xanthomonadales
Family
Acetobacteraceae
Bacillaceae
Beijerinckiaceae
Burkholderiaceae
Caulobacteraceae
Chthoniobacteraceae
Comamonadaceae
Corynebacteriaceae
Dermabacteraceae
Enterobacteriaceae
Fusobacteriaceae
Halomonadaceae
Lachnospiraceae
Lactobacillaceae
Micrococcaceae
Moraxellaceae
Nannocystaceae
Neisseriaceae
Oxalobacteraceae
Pasteurellaceae
Peptostreptococcales-Tissierellales
Porphyromonadaceae
Prevotellaceae
Propionibacteriaceae
Pseudomonadaceae
Rhizobiaceae
Sphingomonadaceae
Staphylococcaceae
Streptococcaceae
Succinivibrionaceae
Weeksellaceae
Xanthobacteraceae
Genus
Acidovorax
Acinetobacter
Anaerococcus
Bacillus
Brachybacterium
Brevundimonas
Burkholderia-Caballeronia-Paraburkholderia
Chthoniobacter
Cloacibacterium
Comamonas
Corynebacterium
Cutibacterium
Diaphorobacter
Escherichia-Shigella
Family_Comamonadaceae
Family_Enterobacteriaceae
Family_Halomonadaceae
Family_Rhizobiaceae
Fusobacterium
Halomonas
Klebsiella
Micrococcus
Neisseria
Peptoniphilus
Polaromonas
Porphyromonas
Pseudomonas
Ralstonia
Rothia
Staphylococcus
Streptococcus
Variovorax
Species
Acinetobacter_junii
Corynebacterium_tuberculostearicum
Cutibacterium_acnes
Family_Comamonadaceae
Family_Enterobacteriaceae
Family_Halomonadaceae
Family_Rhizobiaceae
Genus_Acidovorax
Genus_Anaerococcus
Genus_Brachybacterium
Genus_Brevundimonas
Genus_Burkholderia-Caballeronia-Paraburkholderia
Genus_Chthoniobacter
Genus_Cloacibacteriu
Genus_Comamonas
Genus_Cutibacterium
Genus_Escherichia-Shigella
Genus_Fusobacterium
Genus_Halomonas
Genus_Klebsiella
Genus_Micrococcus
Genus_Neisseria
Genus_Porphyromonas
Genus_Pseudomonas
Genus_Ralstonia
Genus_Staphylococcus
Genus_Streptococcus
Genus_Variovorax
Halomonas_muralis
Rothia_mucilaginosa
Staphylococcus_epidermidis
Staphylococcus_haemolyticus

Supplementary Figure 8: Taxa (phylum to species level) relative abundances for each library as profiled by different 16S rRNA amplicons. Only the 32 most abundant taxa are shown (based on minimum relative abundance in at least one sample).

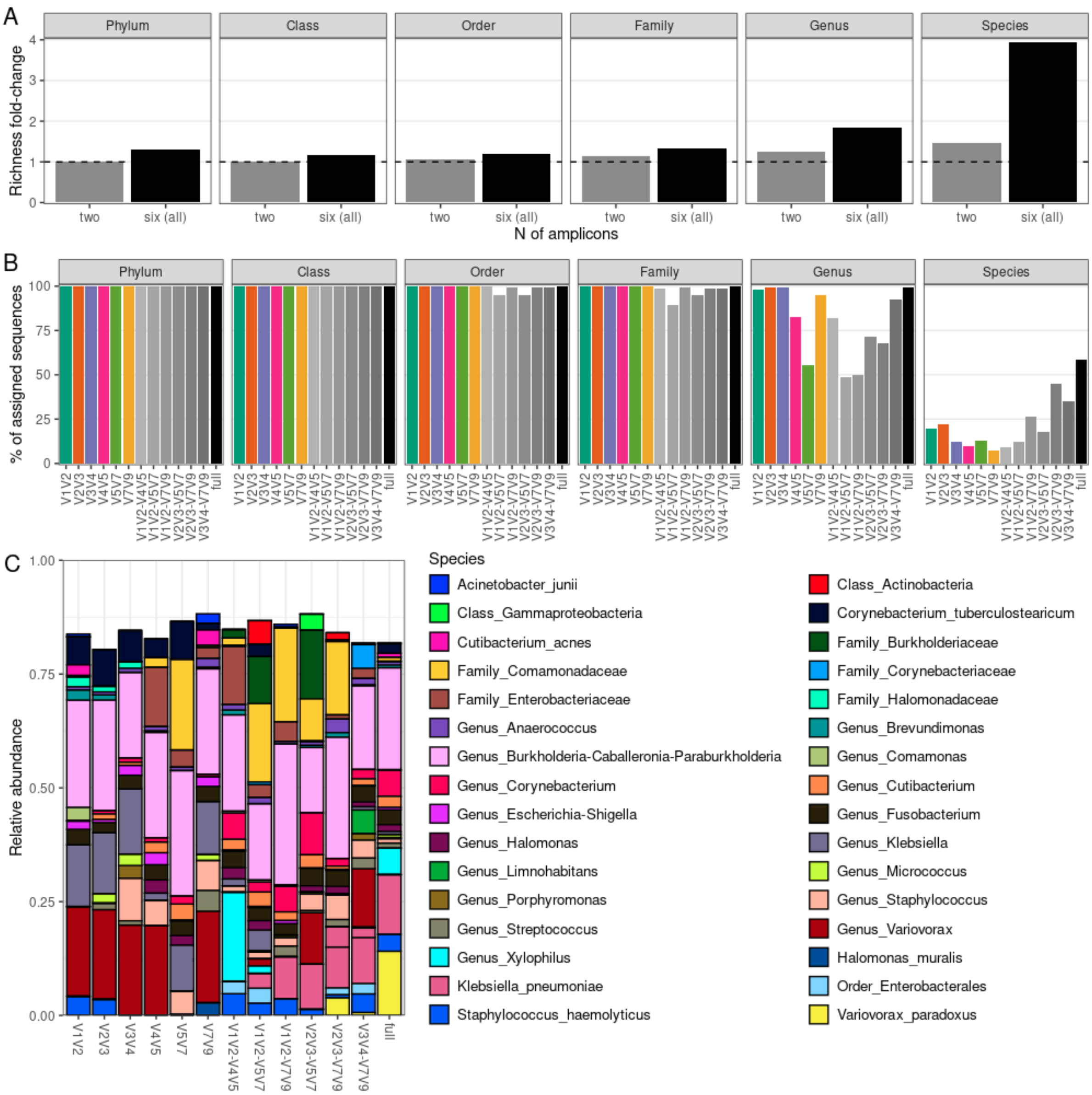

Supplementary Figure 9: Richness and taxonomic composition of Sidle-reconstructed datasets. (A) Fold-increase in taxonomic richness of combinations of two (averaged over all pairs) or six amplicons (as built with Sidle) in comparison with the average taxonomic richness found across amplicon-specific datasets. (B) Percentage of sequences with assigned taxonomy (per taxonomic level) for each amplicon-specific or Sidle-reconstructed dataset (C) Average species relative abundance per amplicon-specific or Sidle-reconstructed dataset. Only the 32 most abundant species are shown (based on minimum relative abundance in at least one sample, which is adjusted for each plot).

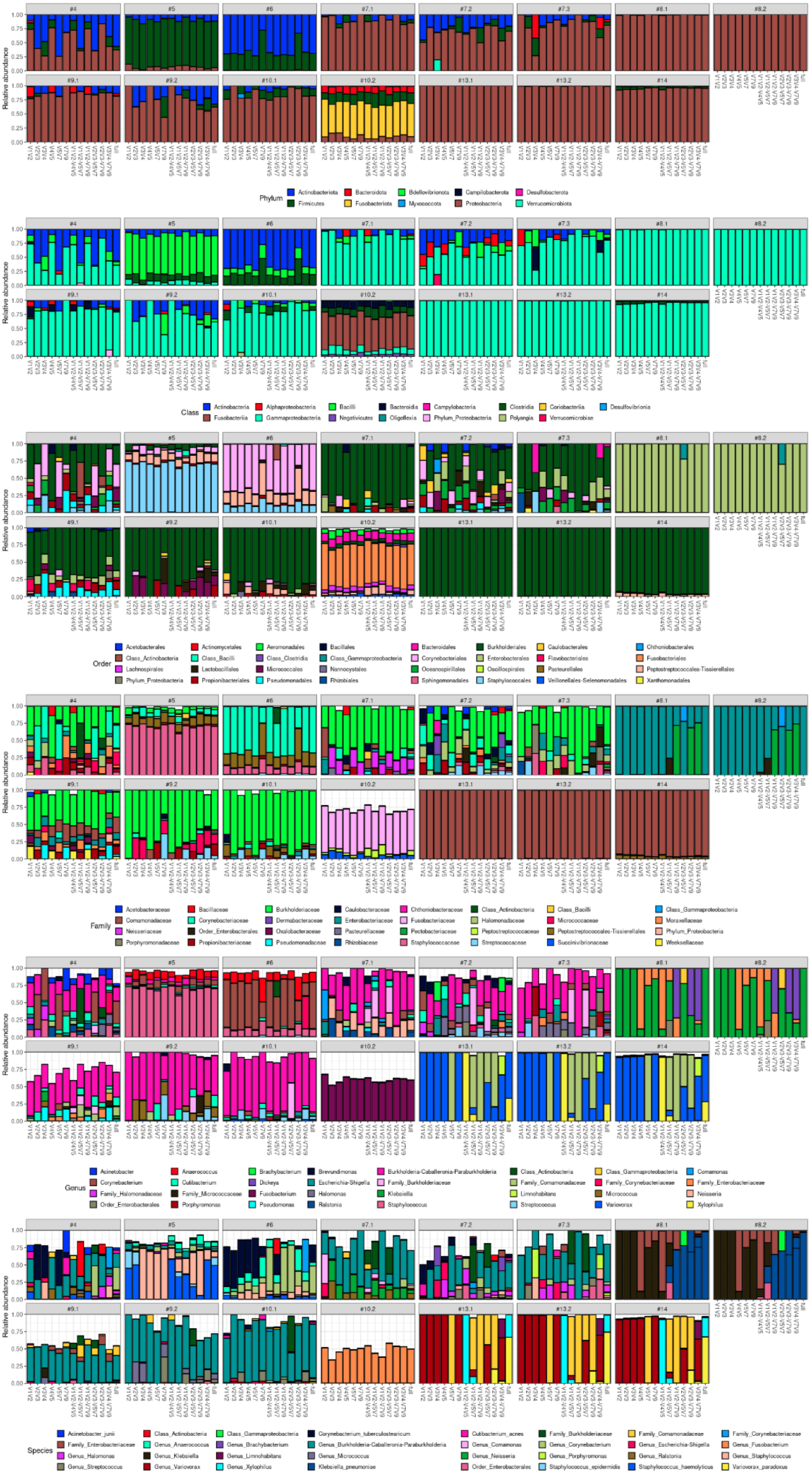

#4
#5
#6
#7.1
#7.2
#7.3
#8.1
#8.2
#9.1
#9.2
#10.1
#10.2
#13.1
#13.2
#14
Relative abundance
0.00
0.25
0.50
0.75
1.00
V1V2
V2V3
V3V4
V4V5
V5V7
V7V9
V1V2-V4V5
V1V2-V5V7
V1V2-V7V9
V2V3-V5V7
V2V3-V7V9
V3V4-V7V9
full
Phylum
Actinobacteriota
Bacteroidota
Bdellovibrionota
Campilobacterota
Desulfobacterota
Firmicutes
Fusobacteriota
Myxococcota
Proteobacteria
Verrucomicrobiota
Class
Actinobacteria
Alphaproteobacteria
Bacilli
Bacteroidia
Campylobacteria
Clostridia
Coriobacteriia
Desulfovibrionia
Fusobacteriia
Gammaproteobacteria
Negativicutes
Oligoflexia
Phylum_Proteobacteria
Polyangia
Verrucomicrobiae
Order
Acetobacterales
Actinomycetales
Aeromonadales
Bacillales
Bacteroidales
Burkholderiales
Caulobacterales
Chthoniobacterales
Class_Actinobacteria
Class_Bacilli
Class_Clostridia
Class_Gammaproteobacteria
Corynebacteriales
Enterobacterales
Flavobacteriales
Fusobacteriales
Lachnospirales
Lactobacillales
Micrococcales
Nannocystales
Oceanospirillales
Oscillospirales
Pasteurellales
Peptostreptococcales-Tissierellales
Phylum_Proteobacteria
Propionibacteriales
Pseudomonadales
Rhizobiales
Sphingomonadales
Staphylococcales
Veillonellales-Selenomonadales
Xanthomonadales
Family
Acetobacteraceae
Bacillaceae
Burkholderiaceae
Caulobacteraceae
Chthoniobacteraceae
Class_Actinobacteria
Class_Bacilli
Class_Gammaproteobacteria
Comamonadaceae
Corynebacteriaceae
Dermabacteraceae
Enterobacteriaceae
Fusobacteriaceae
Halomonadaceae
Micrococcaceae
Moraxellaceae
Neisseriaceae
Order_Enterobacterales
Oxalobacteraceae
Pasteurellaceae
Pectobacteriaceae
Peptostreptococcaceae
Peptostreptococcales-Tissierellales
Phylum_Proteobacteria
Porphyromonadaceae
Propionibacteriaceae
Pseudomonadaceae
Rhizobiaceae
Staphylococcaceae
Streptococcaceae
Succinivibrionaceae
Weeksellaceae
Genus
Acinetobacter
Anaerococcus
Brachybacterium
Brevundimonas
Burkholderia-Caballeronia-Paraburkholderia
Class_Actinobacteria
Class_Gammaproteobacteria
Comamonas
Corynebacterium
Cutibacterium
Dickeya
Escherichia-Shigella
Family_Burkholderiaceae
Family_Comamonadaceae
Family_Corynebacteriaceae
Family_Enterobacteriaceae
Family_Halomonadaceae
Family_Micrococcaceae
Fusobacterium
Halomonas
Klebsiella
Limnohabitans
Micrococcus
Neisseria
Order_Enterobacterales
Porphyromonas
Pseudomonas
Ralstonia
Staphylococcus
Streptococcus
Variovorax
Xylophilus
Species
Acinetobacter_junii
Class_Actinobacteria
Class_Gammaproteobacteria
Corynebacterium_tuberculostearicum
Cutibacterium_acnes
Family_Burkholderiaceae
Family_Comamonadaceae
Family_Corynebacteriaceae
Family_Enterobacteriaceae
Genus_Anaerococcus
Genus_Brachybacterium
Genus_Burkholderia-Caballeronia-Paraburkholderia
Genus_Comamonas
Genus_Corynebacterium
Genus_Escherichia-Shigella
Genus_Fusobacterium
Genus_Halomonas
Genus_Klebsiella
Genus_Limnohabitans
Genus_Micrococcus
Genus_Neisseria
Genus_Porphyromonas
Genus_Ralstonia
Genus_Staphylococcus
Genus_Streptococcus
Genus_Variovorax
Genus_Xylophilus
Klebsiella_pneumoniae
Order_Enterobacterales
Staphylococcus_epidermidis
Staphylococcus_haemolyticus
Variovorax_paradoxus

Supplementary Figure 10: Taxa (phylum to species level) relative abundances for each library as profiled by different 16S rRNA amplicons and Sidle-reconstructed combinations. Only the 32 most abundant taxa are shown (based on minimum relative abundance in at least one sample).

1.2. Supplementary Tables

Patient	Condition	Samples
#1	BPH	#1
#2	BPH	#2
#3	BPH	#3
#4	BPH	#4
#5	BPH	#5
#6	BPH	#6
#7	NMIBC	#7.1, #7.2, #7.3
#8	NMIBC	#8.1, #8.2
#9	NMIBC	#9.1, #9.2
#10	NMIBC	#10.1, #10.2
#11	NMIBC	#11.1*, #11.2*
#12	NMIBC	#12.1*, #12.2*
#13	NMIBC	#13.1, #13.2
#14	BPH	#14
SC	-	#15
NC	-	#16

Supplementary Table 1: Patients and samples included in the study. Samples not sequenced due to low target sequence concentration are indicated with an asterisk. NMIBC patients had two or three samples collected longitudinally during the treatment course. BPH, benign prostatic hyperplasia; NMIBC, non-muscle invasive bladder cancer; SC, Smart Control; NC, DNA extraction negative control.

Amplicon	Primers F	Primers R	Length (F/R)	%GC (F/R)	DI (F/R)	Names (F/R)	Amplicon length
ITS	CTTGGTCATTTAGAGGAAGTAA	GCTGCGTTCTTCATCGATGC	22/20	36/55	0/0	ITS1F/ITS2	230
V1V2	AGRGTTTGATYMTGGCTC	CTGCTGCCTYCCGTA	18/15	39-56/60-67	3/1	27F/357R	330
V2V3	GGCGNACGGGTGAGTAA	WTTACCGCGGCTGCTGG	17/17	59-65/65	3/1	104F/518R	414
V3V4	CCTACGGGNGGCWGCAG	GACTACHVGGGTATCTAATCC	17/21	71-76/43-52	4/4	341F/805R	464
V4V5	GTGYCAGCMGCCGCGGTAA	CCGYCAATTYMTTTRAGTT	19/19	63-74/26-47	2/4	515F/926R	411
V5V7	GGATTAGATACCCBRGTAGTC	ACGTCRTCCCCDCCTTCCTC	21/20	43-52/60-70	3/3	785F/1175R	390
V7V9	YAACGAGCGMRACCC	TACGGYTACCTTGTTAYGACTT	15/22	53-73/36-45	3/2	1115F/1492R	377

Supplementary Table 2: List of primers used in the first-round of PCR. The GC content percentage (%GC) is shown as a range when primers contain degenerate bases. The degeneracy index (DI) of each primer is calculated as the sum of the number of alternate base-pairings for each degenerate base. F, forward primers; R, reverse primers.

Dataset	Total reads	Median length (F/R)	25th Q (F/R)	50th Q (F/R)	75th Q (F/R)	Q-filtered	Denoised	Merged	Non chimeras I	Non chimeras II	Bacterial	Non contaminants	% Total reads decrease	Median ASV length
V1V2	750,924	252/256	36/17	38/25	38/32	694,464	693,540	684,542	667,414	628,279	623,703	614,973	18,1	322
V2V3	801,072	253/255	37/17	38/25	38/32	716,385	715,424	710,670	673,719	647,648	641,088	624,939	22,0	380
V3V4	668,509	259/254	34/19	38/27	38/33	575,366	574,457	569,210	552,435	552,012	496,112	488,560	26,9	409
V4V5	1,674,525	253/249	35/15	38/23	38/30	1,491,565	1,490,588	616,615	614,176	613,575	484,693	477,043	71,5	373
V5V7	881,612	250/251	38/16	38/24	38/30	796,138	794,606	787,603	775,057	768,817	768,676	766,525	13,1	369
V7V9	749,534	255/249	37/17	38/25	38/32	673,733	672,883	668,883	663,163	660,085	655,224	634,974	15,3	377
Total	5,526,176					4,947,651	4,941,498	4,037,523	3,945,964	3,870,416	3,669,496	3,607,014		

Supplementary Table 3: Reads generated per amplicon-specific dataset. Metrics on the raw read data and the number of reads retained after each pipeline step are indicated. Only urine sample libraries are considered. Q-score (Q) percentiles are based on the position defined by the last base of the median read. Non-chimeras I and II refers, respectively, to the *de novo* chimera-filtering step within DADA2 and to the *reference-based* chimera-filtering step with VSEARCH. F, forward reads; R, reverse reads; Q-filtered, reads retained after quality-filtering.

Taxonomic rank	Spearman ρ	P-value
Phylum	0,65	0,16
Class	0,65	0,16
Order	-0,41	0,42
Family	-0,26	0,62
Genus	-0,26	0,66
Species	-0,58	0,23

Supplementary Table 4: Spearman correlation between taxonomic richness and the median ASV length per 16S rRNA amplicon-specific dataset.

Genus	Average RA (%)	Presence in urine	Presence in humans
Comamonas	2,86	Liu et al., 2020	-
Herbaspirillum	0,50	Wu et al., 2018	-
Chryseobacterium	0,27	Zhang et al., 2021	-
Ezakiella	0,22	Qin et al., 2021	-
Facklamia	0,08	Mostafa et al., 2019	-
Mycoplasma	0,04	Smolec et al., 2021	-
Oribacterium	0,01	No	Sizova et al., 2014
Salipaludibacillus	<0,01	No	No
Chromohalobacter	<0,01	No	No
Tepidimonas	<0,01	Richter et al., 2022	-
Clostridium_sensu_stricto_1	<0,01	Mansour et al., 2020	-
Arcanobacterium	<0,01	Ben Khedher et al., 2021	-
Lactococcus	<0,01	Newby and Ramesh, 2014	-
Jeotgalibaca	<0,001	No	Ni et al., 2020
Alkalibacterium	<0,001	No	No
Sum	3,99	-	-

Supplementary Table 5: List of genera detected exclusively in the V1V2 dataset. The average relative abundance (RA) over all samples is shown for each genus. References on the presence of these genera in urine samples or humans are provided.

2. Supplementary References

Ben Khedher, M., Lo, C. I., Diop, K., Morand, A., Armstrong, N., Raoult, D., et al. (2021). Taxonogenomics description of Arcanobacterium urinimassiliense sp. nov., a new bacterial species isolated from urine sample. *New Microbes New Infect.* 41, 100854. doi:10.1016/j.nmni.2021.100854.

Liu, F., Ling, Z., Tang, C., Yi, F., and Chen, Y. Q. (2020). Moderation effects of food intake on the relationship between urinary microbiota and urinary interleukin-8 in female type 2 diabetic patients. *PeerJ* 8, e8481. doi:10.7717/peerj.8481.

Mansour, B., Monyók, Á., Makra, N., Gajdács, M., Vadnay, I., Ligeti, B., et al. (2020). Bladder cancer-related microbiota: examining differences in urine and tissue samples. *Sci. Rep.* 10, 11042. doi:10.1038/s41598-020-67443-2.

Mostafa, H. H., Taffner, S. M., Wang, J., Malek, A., Hardy, D. J., and Pecora, N. D. (2019). Genome Sequence of a Facklamia hominis Isolate from a Patient with Urosepsis. *Microbiol. Resour. Announc.* 8, e00100-19. doi:10.1128/MRA.00100-19.

Newby, B., and Ramesh, K. K. (2014). Urinary Tract Infection in a Preterm Neonate Caused by Lactococcus lactis. *Can. J. Hosp. Pharm.* 67, 453–454. doi:10.4212/cjhp.v67i6.1409.

Ni, Q., Ye, Z., Wang, Y., Chen, J., Zhang, W., Ma, C., et al. (2020). Gut Microbial Dysbiosis and Plasma Metabolic Profile in Individuals With Vitiligo. *Front. Microbiol.* 11, 592248. doi:10.3389/fmicb.2020.592248.

Qin, J., Shi, X., Xu, J., Yuan, S., Zheng, B., Zhang, E., et al. (2021). Characterization of the Genitourinary Microbiome of 1,165 Middle-Aged and Elderly Healthy Individuals. *Front. Microbiol.* 12, 673969. doi:10.3389/fmicb.2021.673969.

Richter, H. E., Carnes, M. U., Komesu, Y. M., Lukacz, E. S., Arya, L., Bradley, M., et al. (2022). Association between the urogenital microbiome and surgical treatment response in women undergoing midurethral sling operation for mixed urinary incontinence. *Am. J. Obstet. Gynecol.* 226, 93.e1-93.e15. doi:10.1016/j.ajog.2021.07.008.

Sizova, M. V., Muller, P. A., Stancyk, D., Panikov, N. S., Mandalakis, M., Hazen, A., et al. (2014). Oribacterium parvum sp. nov. and Oribacterium asaccharolyticum sp. nov., obligately anaerobic bacteria from the human oral cavity, and emended description of the genus Oribacterium. *Int. J. Syst. Evol. Microbiol.* 64, 2642–2649. doi:10.1099/ijs.0.060988-0.

Smolec, D., Ekiel, A., Kłuciński, P., and Kawecki, J. (2021). Occurrence of urogenital mycoplasmas in men with the common genitourinary diseases. *Braz. J. Microbiol. Publ. Braz. Soc. Microbiol.* 52, 2013–2019. doi:10.1007/s42770-021-00620-1.

Wu, P., Zhang, G., Zhao, J., Chen, J., Chen, Y., Huang, W., et al. (2018). Profiling the Urinary Microbiota in Male Patients With Bladder Cancer in China. *Front. Cell. Infect. Microbiol.* 8, 167. doi:10.3389/fcimb.2018.00167.

Zhang, Y., Li, D., Yang, Y., Su, J., Xu, X., Wang, M., et al. (2021). Clinical and molecular characteristics of Chryseobacterium indologenes isolates at a teaching hospital in Shanghai, China. *Ann. Transl. Med.* 9, 668. doi:10.21037/atm-21-933.
